# Interventions to improve inhaler technique for people with asthma

**DOI:** 10.1017/S1463423619000501

**Published:** 2019-08-15

**Authors:** Daksha Trivedi

**Affiliations:** 1 Centre for Research in Public Health and Community Care, University of Hertfordshire, Hatfield, UK

**Keywords:** asthma, clinical outcome, inhaler, interventions, quality of life

## Review question

Does improving inhaler technique improve clinical outcomes and safety in adults and children with asthma?

## Relevance to primary care and nursing

Primary health care professionals including specialist nurses have a vital role in the diagnosis, management and monitoring of asthma in adults and children (National Institute for Health and Clinical Excellence, 2017).

## Characteristics of the evidence

This Cochrane review contains 29 randomised controlled trials (RCT) involving 2210 participants (range 21–201) (Normansell *et al*., 2017). Included studies targeted adults and adolescents (aged 12 years and over) and children (aged under 12 years) with asthma. Follow-up ranged from 2 to 26 weeks. Participants with other respiratory comorbidities, including chronic obstructive pulmonary disease and bronchiectasis, were excluded. Studies examined interventions conducted in hospital, primary care, community or outpatient care settings. These included interventions aimed at improving inhaler technique compared with usual care with no additional intervention, or with alternative interventions such as asthma education only or a different type/intensity of inhaler technique intervention. Twelve studies evaluated enhanced face-to-face training, nine used multimedia-delivered training and the rest used technique feedback devices.

Studies were conducted in Europe (*n* = 11, of which 6 were in UK and 1 each in Belgium, Denmark, France, Ireland and Italy), USA (*n* = 7), Australia (*n* = 4), Asia (*n* = 3), Africa (*n* = 1) and 3 were of unknown origin. Interventions were delivered by trained health care professionals including nurses, respiratory or physiotherapists, pharmacists and physicians.

## Summary of key evidence

Most studies were at high risk of performance and detection bias, as blinding is often not possible with such interventions. Pooled evidence was of low quality overall judged using The Grading of Recommendations Assessment, Development and Evaluation (GRADE), mostly due to concerns about risk of bias and imprecision of the effect estimates. There was considerable heterogeneity between the studies.

Primary outcomes: inhaler technique*, asthma control, exacerbations requiring oral corticosteroids

*Inhaler technique was measured with a standard or validated checklist, or objective measures (eg, peak inspiratory flow (PIF))

Secondary outcomes: quality of life, adverse events, unscheduled visits to health care provider, non-attendance at school or work.

Continuous data were summarised as mean differences (MD) or standardised mean differences (SMD), if different scales were combined, along with 95% confidence intervals (CI). Dichotomous data were summarised as odds ratio (OR) with 95% CI. Findings are summarised for outcomes where data were available and reported.

1. Enhanced inhaler technique education versus control or usual care:

**Correct inhaler technique**. Adults: Face-to-face verbal training increased the number of adults with correct technique compared to controls (three studies, *n* = 258; OR 5.00, 95% CI 1.83–13.65, moderate-quality evidence). Enhanced inhaler technique education also improved technique in most studies as measured by checklist scores, but the variety of checklists used meant the results could not be combined.

Children: Evidence from two studies reported no clear difference in the odds of having correct technique after the intervention, but the result was uncertain. However, one small study of young children (< = 5 years old), trained in inhaler use, reported benefit on PIF in favour of the educational intervention, maintained at two weeks (MD 7.60 L/min, 95% CI 1.43–13.77).

**Asthma control score**. Adults: The intervention showed a favourable non-significant effect (two studies, *n* = 247; SMD 0.48, 95% CI −0.29 to –1.24, low-quality evidence). Two small studies reported ‘complete control’ favouring the intervention (OR 3.18, 95% CI 1.47–6.88; *n* = 134, low-quality evidence).

No significant effects were reported on exacerbations (one study) or quality of life (two studies), but data were too sparse to draw a conclusion. No results could be analysed for other secondary outcomes.

1. Multimedia-delivered inhaler training

**Correct inhaler technique**. Adults: Evidence from two studies showed a non-significant favourable effect on ‘global improvement in technique’ (OR 2.15, 95% CI 0.84–5.50, *n* = 164, moderate-quality evidence).

Two studies, not pooled, reported an increase in MD between the groups on different scales suggesting a benefit of the intervention on the inhaler technique score.

Children: Two studies in children, not pooled, showed some benefits of the intervention when inhaler technique was assessed one month after the intervention using a checklist.

**Asthma control**. Children: There was no significant effect from one study (low-quality evidence). No studies reported any other outcomes of interest.

1. Technique feedback devices

**Correct inhaler technique**. Adults: Two studies, not pooled, reported benefit in favour of the intervention in terms of participants achieving the correct inhalation technique. In one study, the effect was imprecise (OR 18.26, 95% CI 2.22–150.13, *n* = 71; low-quality evidence) and the other study showed a significant effect (OR 4.80, 95% CI 1.87–12.33, *n* = 97; low-quality evidence).

Children: There is no clear benefit of the intervention on PIF rate from two studies (low-quality evidence).

**Asthma control:** There was no evidence of a significant effect in adults (one study) and children (two studies).

**Quality of life**. Adults: No clear difference was reported by two studies using an asthma quality of life scale. One study reported benefit in a quality-of-life ‘responder analysis’ with an imprecise estimate (OR 5.29, 95% CI 1.76–15.89, *n* = 71; moderate-quality evidence).

Children: There was no clear effect from three studies, although mean scores were better in the intervention group (low-quality evidence). Other outcomes were not reported or unsuitable for analysis.

## Implications for practice

Some interventions improve inhaler technique, but this does not clearly translate into improvement in clinical outcomes. Whilst clinicians are required to check their patients’ inhaler techniques, there is no strong evidence to suggest which, if any, interventions are most effective.

## Implications for research

High-quality methodologically sound trials are required with adequate sample sizes, longer duration and standardised measurement methods to detect clinical improvement and adverse effects. Studies need to include economic evaluations and report adherence and its impact on benefit. Larger trials with robust methods to assess the impact of a specific intervention on clinical outcomes are required.
